# The Role of Blockchain Technology in Promoting Traceability Systems in Agri-Food Production and Supply Chains

**DOI:** 10.3390/s23115342

**Published:** 2023-06-05

**Authors:** Techane Bosona, Girma Gebresenbet

**Affiliations:** Department of Energy and Technology, Swedish University of Agricultural Sciences, P.O. Box 75651 Uppsala, Sweden; girma.gebresenbet@slu.se

**Keywords:** blockchain, Internet of Things, agri-food chain, traceability

## Abstract

Due to recurring food quality and safety issues, growing segments of consumers, especially in developed markets, and regulators in agri-food supply chains (AFSCs) require a fast and trustworthy system to retrieve necessary information on their food products. With the existing centralized traceability systems used in AFSCs, it is difficult to acquire full traceability information, and there are risks of information loss and data tampering. To address these challenges, research on the application of blockchain technology (BCT) for traceability systems in the agri-food sector is increasing, and startup companies have emerged in recent years. However, there have been only a limited number of reviews on the application of BCT in the agriculture sector, especially those that focus on the BCT-based traceability of agricultural goods. To bridge this knowledge gap, we reviewed 78 studies that integrated BCT into traceability systems in AFSCs and additional relevant papers, mapping out the main types of food traceability information. The findings indicated that the existing BCT-based traceability systems focus more on fruit and vegetables, meat, dairy, and milk. A BCT-based traceability system enables one to develop and implement a decentralized, immutable, transparent, and reliable system in which process automation facilitates the monitoring of real-time data and decision-making activities. We also mapped out the main traceability information, key information providers, and challenges and benefits of the BCT-based traceability systems in AFSCs. These helped to design, develop, and implement BCT-based traceability systems, which, in turn, will contribute to the transition to smart AFSC systems. This study comprehensively illustrated that implementing BCT-based traceability systems also has important, positive implications for improving AFSC management, e.g., reductions in food loss and food recall incidents and the achievement of the United Nations SDGs (1, 3, 5, 9, 12). This will contribute to existing knowledge and be useful for academicians, managers, and practitioners in AFSCs, as well as policymakers.

## 1. Introduction

Today, the agriculture industry is under pressure due to the increasing world population and demand for sufficient, safe, and high-quality agri-food products. At the same time, the current food supply chain has become more globalized [1], and the dependency on imported food has increased globally by approximately 50% between the years 2006 and 2020 [2]. The increased distances between the locations of food production and consumption have amplified the concerns of consumers regarding food safety and quality [3,4,5,6,7].

Agri-food supply chains (AFSCs) involve a large number of actors with different roles, demands, and responsibilities. This system renders AFSC management and traceability activities highly complex and challenging tasks [8,9,10]. On the other hand, the issue of food safety and quality is an important topic that requires the development of more effective traceability systems [11,12]. Some of the existing traceability systems are not up-to-date or particularly effective in data sharing and interoperability [13,14]. An ineffective traceability system in an AFSC will lead to economic losses and societal health problems due to food contamination and foodborne diseases [10,11,13,14,15,16]. For instance, food contamination affects approximately 10% of the world’s population each year [17]. While it is difficult to estimate the social and economic impact of food fraud, some sources suggest the economic impact could be USD 10–15 billion dollars annually [18,19].

Due to such food quality and safety issues, consumers require a fast and trustworthy system to retrieve necessary information on food products. For instance, Hong et al. [20] described that food items such as milk and milk products, meat and meat products, fish and seafood, oils and fats, fruit juice, coffee and tea, alcoholic beverages, spices and extracts, sweeteners, cereals and pulses, and organic foods are often exposed to food adulteration risks. Adequate information regarding not only the production but also the processing and distribution of food, especially in meat supply chains where product information is less accessible to consumers, is required [12]. For instance, food spoilage and mishandling result in the waste of approximately 30% and 33% of food, respectively [10]. Product counterfeiting is one of the significant challenges in AFSCs, and the mobilization of all actors involved is required to overcome it [13]. In some food supply chains, there are fake claims of food values, e.g., organic, halal, kosher, etc. [20,21,22]. Therefore, there is a requirement to enhance the integrity of credence claims of food using new technologies such as BCT. However, food fraud detection is primarily acheived based on risk assessment and through laboratory analysis of the food itself. The use of excessive preservatives and hazardous chemicals is a common problem in meat supply chains [23]. In some cases, information loss occurs in complex supply chains [24]. 

Industry 4.0 technologies such as information communication technology (ICT), the Internet of Things (IoT), blockchain technology (BCT), big data, cloud and edge computing, and artificial intelligence (AI) (particularly machine learning), etc., play important roles in providing the agri-food sector with the intelligence required to overcome many challenges and ensure sustainable AFSCs [25,26,27]. ICT facilities are required for the collection, storage, processing, analysis, and more efficient use of data [26,27]. However, ICT is not able to avoid the possibility of bias in the use of data, and distrust may arise among different stakeholders who use the data. Such bias can be avoided by using BCT, which enables data management to be divided among a number of supply chain actors, thus, rendering the system more transparent, immutable, and reliable [28]. The supply chain members can act as nodes in a peer-to-peer (P2P) blockchain network.

### 1.1. Traceability in Agri-Food Supply Chains

The major challenges involved in AFSCs include the following: product loss or theft, adulteration, the illegal sale of terminated (or fake) products, illegal labeling, and difficulty in fulfilling customer requirements. Therefore, the traceability system has become a critical component of modern AFSCs [29]. According to the ISO 22005:2007 standard, food traceability is defined as the “ability to follow the movement of a feed or food through specified stage(s) of production, processing, and distribution” [30]. In Bosona and Gebresenbet [31] (p. 4), food traceability is defined as “part of logistics management that captures, stores, and transmits adequate information about a food, feed, food-producing animal or substance at all stages in the food supply chain so that the product can be checked for safety and quality control, traced upward, and tracked downward at any time required”. 

Information is vital for reducing costs, improving yield and quality (while reducing waste), increasing employees’ productivity, and enhancing customer service. It helps to render the supply chain (and its stakeholders) more competitive in the market [32,33]. Traceability, transparency, and auditability are important features that enable one to control (maintain) food quality and safety and increase customer satisfaction [4,32]. Therefore, innovative logistics information systems in AFSCs should effectively provide the abovementioned features.

In modern businesses, market competition is enacted between supply chains rather than individual companies [34]. In this regard, real-time information availability, safety, and reliability are highly important for food supply chain efficiency [14,35]. Therefore, an effective traceability system that avoids information islands in the AFSC is becoming an important tool that can help to fulfill the requirement for the integration of BCT into traceability systems.

### 1.2. Blockchain Technology and Traceability Systems in AFSCs

The existing more centralized traceability systems with centralized data centers are associated with problems such as single point of failure and data tampering [4,7,9,11] (see Section 4.3). Blockchain is a data structure designed to support the applications of distributed digital ledgers where data are safely stored in chained blocks [17]. Although BCT is widely applied within the financial sector, several other industry sectors consider BCT as an important driver of paradigm shifts, and it has gained increasing attention in recent years [36]. In order to facilitate the application of BCT, international standards are being introduced, e.g., ISO 22739:2020—Blockchain and distributed ledger technologies—Vocabulary [37] and ISO 23257:2022—Blockchain and distributed ledger technologies—Reference architecture [38]. Therefore, it has the potential to establish a flow of trustworthy traceability information among the participants in a supply chain [39,40]. However, there is limited knowledge regarding the means by which to develop a conceptual framework for, design, and implement BCT for product traceability, specifically in complex AFSCs with multi-source heterogeneous information, which turns the traceability system into a multidisciplinary process [4,14].

Real-time monitoring systems are becoming an important aspect of food supply chain logistics [41]. Hence, the application of BCT in AFSCs is essential, as it enables one to create a decentralized, immutable, transparent, reliable, and automated system for real-time monitoring and decision making [15,42]. In the application of digital food traceability systems, IoT tools such as radio frequency identification (RFID) have been implemented in many cases, while the BCT-based traceability system is emerging as a potential effective solution [4,9,13,41]. 

Although specific traceability systems have been developed and implemented in AFSCs, there is no traceability system that can satisfy all the needs of the various food supply chains due to the difficulty of developing a common traceability framework in which traceability systems (for a variety of food product value chains) can operate while preserving the flexibility, scalability, and adaptability of the solutions [43]. Therefore, the development of an efficient and effective traceability system is of great importance in the agri-food sector. To support such an effort, research on BCT-based traceability systems in the agri-food sector is increasing, and startup companies have emerged in recent years [3,40]. However, there have been only limited reviews on the application of BCT in the agriculture sector [4,9,13,16,21,27,44]: Yadav and Singh [44] reviewed the application of BCT in the agriculture field; Rejeb et al. [21] conducted a systematic review and discussed the potential benefits and challenges of BCT in the food industry; Yadav et al. [27] reviewed major Industry 4.0 technologies and their applications in AFSCs; Antonucci et al. [16] reviewed the applications of BCT in the agri-food sector; Gayialis et al. [13] conducted reviews on traceability approaches for combatting product counterfeiting and, in this regard, the importance of BCT and IoT; Demestichas et al. [9] reviewed the application of BCT for traceability in the agri-food sector. The abovementioned limited reviews provide a general overview of applications of BCT in the agriculture sector, and more synthesis works are required to promote the application of BCT, which has a promising potential to tackle challenges in complex AFSCs. Review works that focus on BCT-based traceability of agricultural goods are still missing. The current study bridges this gap by reviewing papers that focus on traceability systems with integrated BCT.

The application of BCT-based traceability is still in its infancy, and no well-established and affordable commercial application has been developed to date [3,18,40,45,46]; therefore, a greater knowledge base is required. For instance, there is a limited understanding of the data structure requirements and design of supply chains needed for the effective application of BCT in AFSCs [47]. Moreover, the potential impacts of the application of BCT-based traceability systems in AFSCs are not well-understood [40]. Therefore, synthesizing the available research results and introducing innovative concepts to support the further development and application of effective BCT-based traceability systems in the agri-food sector are highly important tasks [9]. The current paper makes important contributions in this regard.

### 1.3. Objective

In this study, we investigate BCT-based traceability systems in AFSCs using existing relevant studies. The specific objectives of this paper include the following:

- Mapping out the main traceability information in AFSCs, focusing on food value chains which are essential for (re)designing traceability systems that integrate BCT. This facilitates the design and implementation of effective BCT-based traceability systems in AFSCs;
- Reviewing the relevant studies on BCT-based traceability systems in AFSCs to identify, synthesize, and discuss the potential benefits and challenges and provide innovative ideas for the improvement of traceability systems;
- Identifying and discussing the potential implications of BCT-based traceability for the sustainable development of the agri-food sector and areas of focus for future research.

This study offers valuable contributions to the literature focusing on the application of BCT for traceability systems in AFSCs. Firstly, it identifies and discusses the main traceability information, key information providers, and challenges and benefits of BCT-based traceability systems in AFSCs. Then, it illustrates the implications of the implementation of BCT-based traceability systems in a comprehensive way; these help to design, develop, and implement more robust and smart traceability systems, which, in turn, will contribute to the transition to smart AFSC systems. The remaining part of this paper is outlined below. The research methodology is described in Section 2. Section 3 describes the blockchain network and framework for BCT-based TSs. Section 4 presents traceability systems in AFSCs, while Section 5 discusses the implications of BCT-based traceability systems. Finally, Section 6 presents the conclusion. 

## 2. Methodology

**Figure 1:**
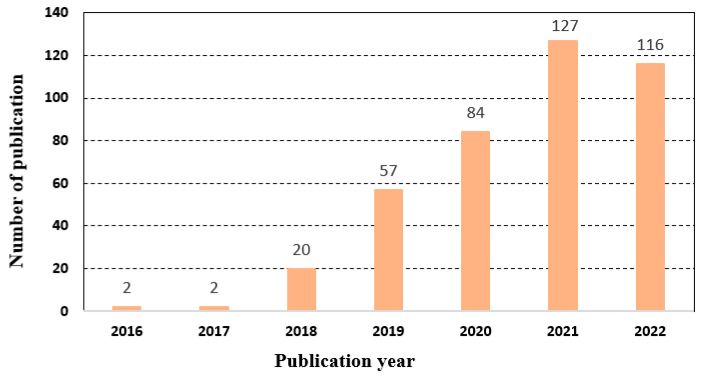
Literature search results using the search string.

This study is based on literature retrieved from the Scopus database. The final literature search was performed on 22 September 2022. The Scopus database was used because it contains high-quality publications from Elsevier and thousands of other international publishers of scientific papers. The search was performed targeting the combined fields of the article title, abstract, and keywords for each publication without limiting the publication duration. The appropriate keywords and Boolean operators were first identified and then used to create a search string with the combined terms (“Agri-food” OR “food”) AND “blockchain” AND “traceability”. In total, 408 publications were retrieved from the years 2016 to 2022. No publication from before the year 2016 was retrieved for years, indicating that studies on BCT-based traceability in AFSC have emerged recently but are growing rapidly. Figure 1 illustrates the increase in the number of scientific publications on BCT applications for traceability in AFSCs in recent years. 

Out of 408 papers, the 59 most relevant peer-reviewed papers were identified and used in the final review. This identification was performed by reading the titles, abstracts, and conclusions of the articles. Articles written in the English language with a focus on BCT-based traceability systems were selected and used in the final review. In addition, 8 additional peer-reviewed papers and 11 documents were used, making the total number of references used in this paper 78.

**Figure 2:**
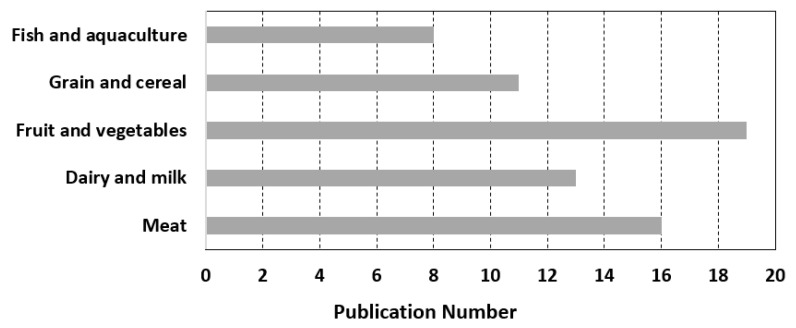
Illustration of focal areas of BCT-based traceability systems in the retrieved papers.

In order to understand the focal areas of the retrieved articles, five value chains were identified: meat; dairy and milk; fruit and vegetables; grain and cereal; and fish and aquaculture. Then, papers focusing on each of these value chains were identified (see Figure 2). Relatively speaking, the existing BCT-based traceability systems focus more on fruit and vegetables, meat, and dairy and milk, as illustrated in Figure 2. However, most of the papers discuss traceability systems using BCT in the agri-food sector in general terms, without a specific area of focus. In this regards, there is a limitation due to the fact that there could be papers not captured in this review.

## 3. Blockchain Networks and Frameworks for BCT-Based Traceability Systems

### 3.1. Distributed Digital Ledger

**Figure 3:**
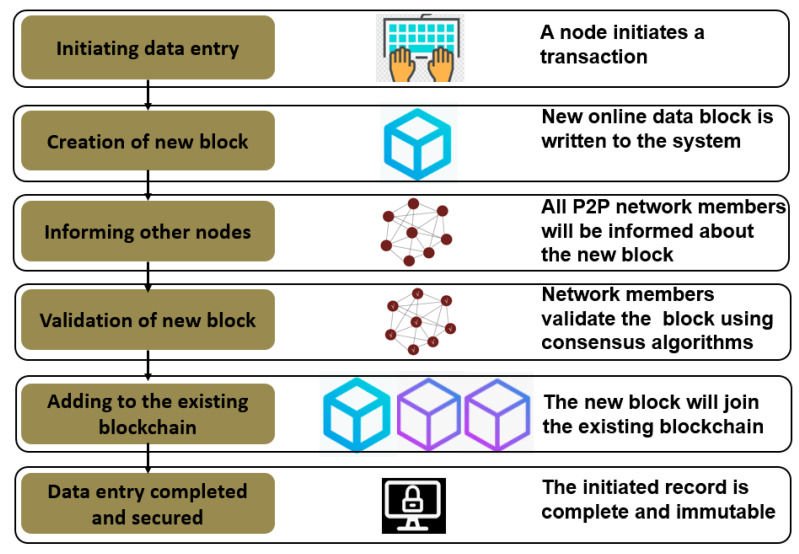
Creation process of a new data block in an existing blockchain network.

Blockchain is an immutable and distributed digital ledger containing chained data blocks [9,11,13,39] (see Figure 3). It is a ledger with a growing list of data records that are validated by the P2P network members/nodes [9]. In BCT, a chain of data is created by immutably linking a new block with the previous block. Once data have entered the Blockchain, no one can alter them, as an attempt to corrupt the data in one of the blocks will render the following blocks invalid. This property of BCT enables one to tackle data modification problems. For instance, in traditional AFSCs, the modification of travel histories, ingredients, expiry dates, etc., by intermediaries, is one of challenges in the food industry [11]. 

Unlike a centralized system, there is no centralized control in a BCT-based system, and all negotiations among the users (nodes) are mediated by consensus mechanisms [17]. To increase the reliability of digital data, BCT enables one to verify the data on three levels: collection, storage, and validation [36,48]. In BCT, a P2P distributed network stores data at different nodes, and data can be transferred between blockchain clients (nodes) [9,49]. 

### 3.2. Components of Blockchain System

**Figure 4:**
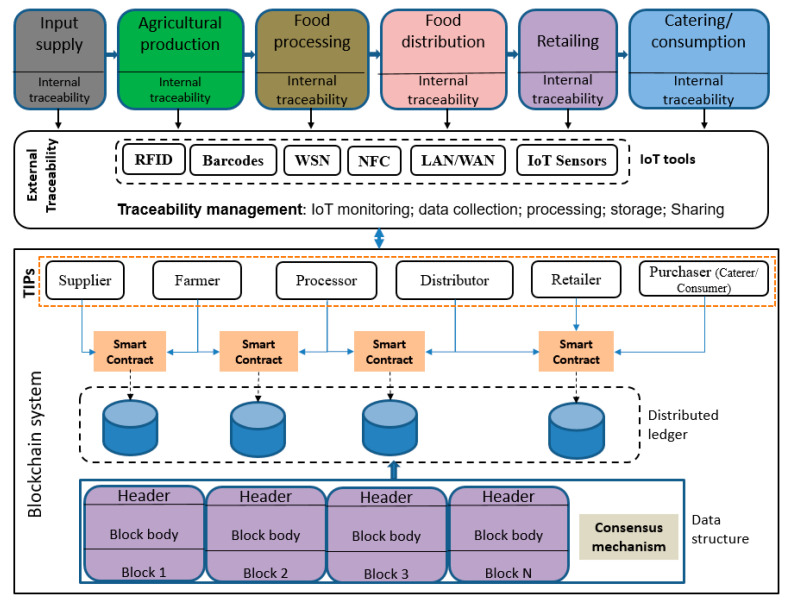
Simplified presentation of a BCT-based traceability system for AFSCs. TIPs—traceability information providers.

Blockchain is a “distributed ledger with confirmed blocks organized in an append-only, sequential chain using cryptographic links” [37]. The main components of blockchain include the following:

**Node**—a computer with a copy of the blockchain data ledger that is operated by a P2P network member in the blockchain environment;

**Transaction**—a record containing data from a responsible owner who wishes to add that record to the blockchain in an immutable manner [17]. Each transaction must be validated before its final integration into the blockchain; 

**Block**—a structured data compartment with data that belong to the block. Each block has a block header and a block body (see Figure 4). The block header defines the current block and its hash value, the hash of the previous block, the timestamp, and the block number. The block body defines the data structure that stores all the verified transactions in relation to the creation of a specific block [9,14,50]; 

**Consensus mechanism**—a set of rules and procedures that enable network nodes reach a consensus and perform blockchain operations;

**Smart contract**—is defined by Szabo [51] (p. 1) as “a computerized transaction protocol that executes the terms of a contract”. The application of BCT may be based on certification access or smart contract mechanisms [37]. In supply chains, approaches based on ‘smart contracts’ are more promising, as they enable peers in the network to have full visibility [9]. Each node of the P2P network has access to the history of all the smart contracts and transactions, as well as the current state of all the smart contracts. For instance, the Ethereum blockchain is a typical blockchain designed to support smart contract implementation and is suitable for traceability systems [9,41]. Yao and Zhang [29] developed and implemented an Ethereum blockchain with less data storage pressure and without the leakage of sensitive data belonging to each participating company. However, for large-scale networks, the Ethereum blockchain has drawbacks, such as the difficulty of protecting sensitive data, and inefficiency in terms of time and costs [41].

### 3.3. Block Creation Process

A new data block can be created and enter an existing blockchain in six steps. First, a node in the P2P network initiates a transaction (e.g., new data entry). Then, a new data block will be created, and the creation of this new block will be communicated to the remaining nodes in the blockchain for validation (see Figure 3). After the transaction is validated by the other nodes, the new block will join the existing chain of blocks. Finally, the initiated transaction will be successfully accomplished and secured. When the block creation process is complete, the data at all the nodes in the P2P network will be updated with the newly added data block [41]. Figure 3 presents the conceptual illustration of the data block creation process. 

### 3.4. Blockchain Consensus Protocols

Consensus protocols are agreements that dictate the operations performed in the blockchain. Consensus protocols enable all the nodes in the P2P network to maintain the same distributed digital ledger [52]. The main available blockchain consensus protocols are described in the following subsections:

**Proof of work:** Proof of work (PoW) is of greater benefit to those who own better equipment and have a higher chance of creating the next block due to their higher hash rate. This may cause the BC to become more centralized (when it needs to become more decentralized) [9]. PoW is secure but computationally expensive and energy-intensive [39,49]; 

**Proof of stake:** Proof of stake (PoS) protocol is more decentralized than PoW and enables one to address the limitations of the PoW protocol in relation to the decentralization issue [9]. PoS is also cost- and energy-efficient [39]. However, if a user or a group of users obtain 51% of the hashing power, this poses a risk of what is known as the 51% attack, which refers to the control and manipulation of the blockchain; 

**Proof of object:** Proof of object (PoO) is a concept according to which the owner of the object establishes the ownership. PoO can be used as an alternative to PoW and PoS; 

**Proof of Activity:** Proof of activity (PoA) is a hybrid consensus mechanism (of PoW and PoS) [11]. PoA increases the security of the blockchain and reduces the probability of a 51% attack to nearly 0%. However, similar to PoW, PoA requires a relatively higher power consumption and more powerful hardware.

### 3.5. Types of Blockchains

From an accessibility point of view, BCTs can be public blockchains (e.g., Bitcoin and Ethereum networks), private blockchains (with invited participants), or permissioned (hybrids of public and private) blockchains [1,9,11]. Anyone can join a public blockchain network, as it does not require permission. A private blockchain network is a closed system to which only predefined peers (or owners) have access, and it helps companies to protect their sensitive data from becoming public. In a permissioned blockchain, in addition to the owners of the network, a user can obtain permission to access the network after completing identity verification and can perform specific activities that he/she is allowed to carry out. BCT-based traceability systems can be implemented in all three of these BCT types [9,36].

## 4. Traceability Systems in AFSCs

### 4.1. Traceability Information

Traceability systems in AFSCs require a large volume of data from the main supply chain actors. Such information comprises both qualitative and quantitative information. Before information collection, the traceability information providers (TIP) must be identified. TIPs are the principal providers of traceability information, i.e., key data elements (KDEs) required regarding critical traceability events (CTEs) throughout the supply chain [8,43]. CTEs are events at known physical points that effectively link the incoming products, their transformation, and outgoing products. KDEs constitute the set of data (e.g., lot numbers, quantities, locations, dates, ingredients, etc., as indicated in Table 1 and Table 2) needed in order to obtain complete traceability information [8]. 

Each TIP is responsible for the accuracy of the specific information entered into the traceability system. Not all the stakeholders in a supply chain are necessarily TIPs, and they may not be responsible for data quality [9]. Therefore, it is important to clearly identify the TIPs when designing the traceability system. In AFSCs, the main TIPs include input suppliers, farmers, processors, distributors, retailers, food caterers, and restaurants (see Figure 4). 

The required traceability information varies from product to product and according to the environment, handling methods, etc. [34]. Therefore, it is important to define the type of traceability information, i.e., the KDE needed from each TIP in the AFSC (see Table 1 and Table 2 and Figure 4). These KDEs can be collected from different stages of the supply chain: the input supply, agricultural production and harvesting, food processing and storage, transportation, retailing, catering and consumption. At the end of the supply chain, retailers should make available all relevant data about the products on sale so that consumers can verify the product history and make purchasing decisions. More detailed information on each stage is provided in Table 2. 

### 4.2. Internet of Things and Traceability Information Handling

Traceability information can be recorded manually or automated using IoT technologies [4,9]. Manual data recording and transfer on paper-based ledgers or computers are subjected to human errors during the inputting of information into the database and have been exposed to result in poor-quality data, resource inefficiency, and information loss [38]. On the other hand, automated data-capturing and data-transferring systems are more efficient and use IoT tools such as RFID, wireless sensors networks (WSNs) (as well as QR codes and barcodes), near-field communication (NFC), wired connection (e.g., LAN/WAN), wireless connection (e.g., Bluetooth/Zigbee), etc. [4,9,11]. For instance, information about the crop growing process and environment can be gathered using sensors that can autonomously store the required data at regular, predefined intervals. RFID is widely used in AFSC management and provides safe information. WSNs complement RFID in cases where wireless sensors are used to capture data on temperature, humidity, sound, pressure, etc., which are transferred for external data storage and use. WSNs are highly important, as they are energy-efficient and battery-powered and use many sensors. 

The captured data can be organized as on-chain data and off-chain data. Off-chain data can be kept at the company (TIP) level and can include confidential data [9]. Information and data gathering, sharing, and usage should be efficient to increase the benefits gained from digitalized systems [47]. The quality of traceability information is also important for quality controllers and purchase decisions made by consumers [58]. 

### 4.3. Traceability System

#### 4.3.1. Internal and External Traceability

Traceability can be described on two levels: internal traceability and external traceability. For instance, in AFSCs, internal traceability focuses on information related to the sources (origins) of ingredients and detailed information on food processing and packaging. It is carried out on the company level, i.e., each actor in the supply chain can carry out internal traceability.

An external traceability system covers the entire supply chain and is also known as a supply-chain-level traceability system. It should be built on information regarding product ingredients, sources, processing, transportation, storage conditions, retailing, and catering service. It enables one to connect information from all the involved actors, reconstruct the product history throughout the entire supply chain, and improve product quality and safety [57]. For instance, Zhang et al. [14] developed a trustworthy external traceability system for grain and food oil supply chains through the integration of all relevant data on agricultural production, processing, distribution, retailing, and consumption levels.

#### 4.3.2. Traceability System with a Centralized Database

The current food value chains are more centralized, monopolistic, asymmetric, opaque, and less secure. The existing traceability systems also depend on centralized data centers. A centralized traceability system has a central computing system using a central database with a central server. It can also use IoT tools for data capture and transfer. The introduction of IoT tools into AFSCs has created opportunities to develop effective traceability systems that use centralized database systems [4,9,36]. IoT tools enable traceability systems to become more reliable, auditable, and transparent [4,36]. Many of the existing digital traceability systems are centralized traceability systems. However, traceability systems with central databases have some problems that need to be addressed. They are built using centralized cloud infrastructures that are often provided by a third party [4,29,36]. This leads to issues related to data integrity and tampering, and single points of failure [7,11,49]. These issues lead to limitations regarding transparency, security, availability, confidentiality, and auditability. In such a centralized system, since the information flow relies more on trust, adequate and honest information is expected from each TIP. Information limitations restrict supply chain participants from accessing and understanding the quality and origin of the product [4]. All actors in the AFSC rely on a single information point. These problems can be better addressed using BCT-based traceability systems. BCT security is strong due to the higher number of layers of security in place, e.g., one level of security, through the use of cryptographic hash, and an additional level of security can be achieved using P2P networks [9]. 

#### 4.3.3. Traceability Systems with BCT-Based Distributed Ledgers

Decentralized traceability systems are emerging as new traceability systems are built with the integration of BCT. BCT can be integrated into traceability systems either by redesigning an existing traceability system or during the development of a new traceability system in an AFSC. The decentralized character, consensus protocol, and process integrity of P2P networks provide BCT with an advantage in addressing challenges involved in product supply chains [13]. It uses a P2P network and distributed digital ledger supported by decentralized data storage and IoT tools for automated data capture and transfer [9,48,49]. In this case, the records are tamper-proof, with increased trustworthiness, as the integrity and flow of information rely not on trust but on the blockchain’s built-in smart contracts. Unlike centralized traceability systems, BCT-based traceability systems have an enhanced data storage capacity due to their distributed P2P networks, and automated records from IoT networks and other transaction events can be traced more effectively [45]. Starting in 2016, different BCT-based traceability systems have been proposed and tested in agri-food supply chains [9].

In the development and application of BCT-based traceability systems, the major actions to be taken include the following: identifying the participants (e.g., stakeholders), ensuring that all the participants (including any IoT devices to be used) are registered as users and can digitally sign each operation on the blockchain ledger, determining the important traceability information in each stage of the entire AFSC, and identifying the data owner and means of data collection and management [9,14]. The collected data should be entered into the system, verified, and standardized through smart contracts before being entered into the blockchain system and stored in chronological order according to the time sequence of the transactions [19,50]. 

### 4.4. Challenges in the Application of BCT-Based Traceability in AFSCs

Although BCT is perceived as an emerging technology that can lead to breakthroughs in product supply chains, there are barriers that hinder its implementation [59]. Rejeb et al. [21] discussed the technical, organizational, and regulatory challenges of the application of blockchain technology in the food supply chain. Global AFSCs involve actors from different parts of the globe. Therefore, making processes in different stages of AFSC management more transparent, auditable, and reliable is a challenge [36], especially when there are limitations regarding data governance. Limitations on data storage capacity, scalability issues, the potential leakage of private sensitive data, and high investment costs are some of the widely reported challenges regarding the application of BCT [21,60]. Table 3 presents a more detailed list of challenges that hinder the development and implementation of BCT-based traceability systems in AFSCs. 

### 4.5. Perceived Benefits of BCT-Based Traceability Systems in AFSCs

Establishing a traceability system with the best performance enables one to not only trace/track product flow but also to improve food quality, economic benefits, and AFSC management [6]. This, in turn, enables one to reduce food loss and waste, logistics costs, and resource demands for AFSC management [26,29,64]. In the agri-food business sector, traceability systems increase the profits of the supply chain actors through cost reductions and increases in asset value [9,39,64].

BCT-based traceability enables one to effectively detect product fraud, points of contamination, and food spoilage; protect the company brand; guarantee the origin of a product; and provide trustworthy information on the ingredients and processing methods of products [16,17,18,46,64]. Table 4 presents some major benefits of BCT-based traceability systems.

The benefits of BCT-based traceability systems in complex AFSC are expected to be maximized with future research on blockchain systems, as well as the development and implementation of innovative designs of such systems [66]. Therefore, future studies could focus on enhancing the scalability (to cover the required number of nodes), data storage capacity, system security (e.g., through developing smart contract methods that are less vulnerable to security attacks), and privacy protection of these systems (avoiding the dissemination of sensitive or personal data) and creating specialized principles and guidelines to facilitate the implementation of BCT in AFSCs with lower system costs [11,60]. The blockchain system can store miscellaneous data, but the traceability system can ensure the collection, storage, and transmission of credible traceability information [14]. 

The infrastructure costs of BCT-based applications are also poorly understood, and in-depth investigations are needed in this regard [63,71]. Recent research on BCT in the agri-food sector has focused on increasing trust and transparency regarding food products. However, future studies on emerging BCT-based traceability systems should also consider potential reductions in cost and food losses, the understanding of marketing systems and supply chain actors’ behaviors in relation to BCT applications, and the development of user-friendly software and functional application tools [61,63]. In the existing traceability systems, the input supply stage is not given much attention. Therefore, future studies on BCT-based traceability systems should focus on determining key traceability information and the collection, storage, and sharing of the information in the input supply and agricultural production stages. Effective data capture (starting from the input supply and agricultural production stages) and recording in blockchain systems could enhance the overall performance of the traceability systems of AFSCs, as this could avoid the possibility of data adulteration. The flow of trustworthy information about the types of inputs (used during agricultural production) and the quality and safety of food products lead to satisfaction among customers and other stakeholders downstream of AFSCs, including food-quality-controlling authorities.

## 5. Further Discussion

### 5.1. Implication of BCT-Based Traceability in AFSC Management

BCT is a disruptive but robust and promising technology that can enable one to tackle many challenges involved in AFSC management [1,11,35,59]. It has the potential to revolutionize the management of the entire supply chain and the way in which each party in the BCT network conducts business [33,59]. It can reduce human interference and costs in AFSC management due to the BCT-based automation of the system supported by multiple IoT sensors that gather data and share these data with cloud servers for processing and decision-making, with less time and resource expenditure [11,35,57,70]. Depending on the pre-specified conditions used in smart contracts implemented in the blockchain, a transaction or process can be triggered automatically. This facilitates the effective automation of AFSC management. However, efforts to develop different BCT-based traceability systems for different AFSCs may be costly. Therefore, it is important to develop BCT-based traceability platforms that can be adapted for a variety of food products with lower investment costs [7]. 

### 5.2. Implications of BCT-Based Traceability for Food Recall Incidents and Management

On the one hand, food contamination events are frequent in food supply chains. On the other hand, the food recall processes used to mitigate health and economic crises are manual and inefficient. For instance, the food recall process in the United States (from the identification of the contaminated product and source of contamination to the taking of actions to mitigate the crisis) takes approximately one week on average [72]. This time-inefficient process leads to more societal and economic crises. This problem is aggravated by the lack of automation and shared data used in the recall process. Even though each actor in the supply chain has their own electronic data-handling system, there is a lack of integration of these data through the entire supply chain. In this regard, BCT-based traceability systems could play an important role in realizing greater information connection and the automation of food recall processes, which, in turn, could reduce costs, illnesses, and deaths related to food contamination.

Understanding the real-time food safety status is important for AFSC actors [11]. This can enhance transparency in the AFSC and customer confidence. For instance, consumers with a strong perception of health risks have greater intention to purchase BC-traceable food products, as BCT-based traceability provides more information on product quality and safety [46].

Effective BCT-based traceability enables reductions in the number of food contamination and related recall incidents, as well as limiting damage to consumer rights and improving the performance of recall processes [4,11,65]. This, in turn, reduces food waste on the retail and consumption levels and the nutritional loss and negative environmental impacts associated with food waste.

Food contamination and fraud are important issues to be addressed in AFSCs [12,64]. A well-designed flow of traceability information enables one to identify the source of food contamination more accurately with less time and resource expenditure [11,14]. For instance, a traceability process that takes approximately one week can be reduced to approximately two and a half seconds with a BCT-based traceability system [44]. 

### 5.3. Implications of BCT-Based Traceability for Reductions in Food Loss and Waste

Food loss is one of the major challenges facing the food sector. Therefore, traceability solutions should be designed in such a way that they support efforts to reduce food loss. One cause of food loss and waste is delayed and inefficient food recall processes. Therefore, a food recall process that uses an automated BCT-based traceability system enables one to immediately alert all concerned actors throughout the supply chain. This, in turn, helps to avoid food waste that may be generated due to delayed food recall processes.

Although digital technologies, e.g., IoT and BCT, can reduce food loss and waste, there is a knowledge gap regarding the extent of FLW reduction in different types of food value chains [26]. This problem requires attention in future work in order to generate knowledge, support the development of policy options, and enhance sustainable food systems [67]. 

### 5.4. Implications of BCT-Based Traceability for the Achievement of SDGs

As the application of BCT expands in many business sectors, it is expected that its contribution to sustainable development will increase [65]. Therefore, effective implementation of BCT-based traceability systems enhances the achievement of the United Nations’ SDGs (see Table 5). BCT supports the transition towards resilient and sustainable food systems [25,47,61]. BCT enhances the sustainability of AFSCs in regard to economic sustainability (through effective traceability with lower costs and efficient information sharing, as well as the automation of AFSC management), environmental sustainability (through effective resource management and reductions in product loss and product recall incidents), and social sustainability by addressing the areas of accountability, trust, food safety, and fraud prevention [59,73]. The application of BCT enables one to collect, store, and use data and information that are vital for monitoring and improving sustainability, including soil, water, and climate conditions in the agricultural production area, pesticide and fertilizer applications, efficient processing and waste reduction, relationships with stakeholders, and human rights [57]. Future studies should also focus on the integrated collection and management of traceability information and data for food quality grading and for monitoring the sustainability performance of AFSCs. 

BCT-based traceability systems can improve AFSC management by increasing food quality, safety, and value while reducing post-harvest food losses and wastes, as well as costs associated with food losses [49,64,74]. These improvements also have implications for efforts to reduce the negative environmental impacts of the agri-food sector and improve the sustainability of AFSCs [33]. However, the use of technologies such as IoT and BCT within the agri-food sector and their impacts on sustainability need to be explored further [36]. Table 5 presents some SDGs which could be positively impacted by BCT-based traceability systems.

**Table 5:** Some of the potential contributions of BCT-based traceability to the achievement of SDGs.

SDG	Description of Potential Contribution to the Achievement of the SDG	Reference
SDG#1: No poverty	It boosts the national and international market for the agri-food sector, which, in turn, helps to increase the household income of farmers and promote SMEs, as well as local and national economies.	[47,75]
SDG#3: Good health and wellbeing	It guarantees food security (through a strong capacity to monitor food quality and safety and minimize food contamination), which is important for societal health and wellbeing.	[47]
SDG#5: Gender equality	It enables one to monitor and improve labor working conditions and enhance fair trade activities.	[46,47]
	It reduces energy consumption and costs which, in turn, can reduce environmental impacts and increase sustainability.	[39,61]
SDG#9: industry, innovation, and infrastructure	It enables one to build innovative and resilient digital infrastructure within AFSCs.	[75]
SDG#12: Sustainable consumption and production	It enables one to reduce food loss and waste through effective information flow that helps consumers to make efficient purchase decisions; ensures fast and effective recall processes; and promotes sustainable consumption.	[47,61]

### 5.5. Implications of Bridging the Digital Divide

BCT has changed how firms collect data and interact with stakeholders and customers, rendering these processes more efficient [40,76]. This makes BC technology more attractive for global food supply chains that involve stakeholders (actors) from different countries. In global food supply chains, the actors can be from industrialized and non-industrialized countries, meaning that there is a digital divide that hinders the application of innovative digital technologies in areas with less digital infrastructure [26,62]. Some research efforts have been undertaken to support the application of BCT in developing countries. For instance, Musah et al. [77] investigated the potential contributions of BCT to the cocoa bean supply chain in Ghana and found that BCT increased the traceability, transparency, and efficiency of the supply chain. Similarly, Tsolakis et al. [47] studied the implementation of BCT in the Thai fish industry and concluded that BCT led to increases in the transparency, immutability, traceability, trust, and sustainability performance of the fish product supply chain.

The implementation of BC-based TS may be costly and challenging. Therefore, the future application of BCT will benefit from digitalization and related training and education programs run by governments and development agents as part of the bridging of the digital gap. Mattila et al. [78] discussed the importance of institutional changes and financial support in harnessing BCT to achieve SDGs in low- and medium-income countries. Government agencies also have an important role in developing policy options that can facilitate the implementation of traceability systems and emerging digital technologies in the agri-food sector. It is expected that BC technology’s implementation in the agri-food sector will increase rapidly. To facilitate such a technological application and increase the benefits of innovative digital technologies (such as BCT, IoT, machine learning, and artificial intelligence), more training and education on digital infrastructure within agriculture organizations is required [16].

## 6. Conclusions

This paper presented the results of study on traceability systems within agri-food supply chains (AFSCs) that integrate emerging blockchain technology (BCT). This was a literature-based study that aimed to identify and discuss the following: the main types of traceability information needed to build effective traceability systems, the benefits and challenges of BCT-based traceability systems, and the potential implications of the application of BCT-based traceability systems within AFSCs. The literature search was conducted using the Scopus database and the search string (“Agri-food” OR “food”) AND “blockchain” AND “traceability”.

The results indicated that the existing traceability systems with the application of BCTs focus more on fruit and vegetables, meat, dairy, and milk. Although research on BCT-based traceability systems within AFSCs has been increasing since 2016, their application in real businesses is in its infancy and is more complex when the AFSC under consideration is global. Therefore, more research needs to be performed in order to design and implement effective BCT-based traceability systems for specific value chains in AFSCs.

Studies have indicated that the existing traceability systems mostly use a centralized database with a central server and computing system that are often provided and controlled by a third party. Such a traceability system has limitations (issues) regarding transparency, trust, security, the availability of information, confidentiality, and auditability. It is difficult to acquire full traceability information from such centralized traceability systems, and there are risks of information loss and data tampering. On the other hand, a BCT-based system renders traceability data easily accessible and immutable. A BCT-based automated traceability system also enables one to develop and implement a decentralized, immutable, transparent, scalable, and reliable system in which the automation of processes facilitates the monitoring of real-time data and decision-making processes. 

In order to promote the development and application of BCT-based traceability systems, the main types of traceability information (key data elements) as well as the key traceability information providers (TIP), challenges, and benefits were mapped out in this study considering the entire AFSC: the input supply; agricultural production; food processing; distribution; retailing; and consumption (including catering and restaurants). The results also indicated that BCT-based traceability systems have important positive implications for the following areas: the automation and improvement of AFSC management; reductions in food recall incidents and the automation of the recall process; reductions in food loss and waste, as well as nutrition, economic, and resource losses related to food loss; the achievement of the United Nations SDGs; and the bridging of the digital divide to facilitate the economic development of low- and medium-income countries and support them in achieving their national SDG targets. 

Although BCT-based traceability systems enable one to address issues such as transparency, trust, product traceability, etc., they are not a universal solution for all issues within complex AFSC management, especially when the supply chain in question is long or global in nature. This study will be useful for academicians, managers, and practitioners involved in AFSCs, helping them to understand and promote the design, development, and implementation of BCT and BCT-based traceability systems in AFSCs, which, in turn, will contribute to the transition to smart AFSC systems.

## Figures and Tables

**Table 1:** Summary of relevant description regarding CTEs, KDEs, and TIPs in AFSCs [53,54].

Major CTEs	Important KDEs	Typical TIPs
Transformation (e.g., production event, packaging event, etc.)	Type, owner, location, date, and time of the event; item ID and type; Batch/Lot/Serial#; quantity and Unit of Measure; Activity ID and Type; supplier identity	Input supplier and processor
Transportation (e.g., shipping and receiving events)	Type, owner, location, date, and time of the event; item ID and type; Batch/Lot/Serial#; quantity and Unit of Measure; Activity ID and Type; supplier identity; Location of Trading Partner	Distributor (transport service provider)
Depletion (e.g., consumption and disposal events)	Type, owner, location, date, and time of the event; item ID and type; Batch/Lot/Serial#; quantity and Unit of Measure	Processor, distributor, Retailer, Food service provider

**Table 2:** Main types of traceability information (data) needed for agri-food product traceability.

AFSC Stage	Traceability Information (KDEs)	Reference
Input supply	Identity (of clients/participants); seed type and amount; fertilizer type and amount; pesticide type and amount; other agrochemicals; feed type and amount.	[55,56]
Agricultural production (farm stage)	Plant food products: producer identity; farm certification; commodity type and variety; geographic origin (growing area name and location); soil conditions; greenhouse conditions (if used) such as air temperature, humidity, water temperature, and pH value; cultivation information; plant growing process; harvesting conditions and method; harvested amount.	[14,36,41]
	Meat and meat products: individual animal identification; feeding conditions; vaccines; genetics; age, sex, weight, and health history of the animal.	[12,55]
	Dairy products: dairy farm; date and time of milk collection; milk quantity and quality; identity of milk collector; milk reception and timestamp.	[39,55]
	Aquaculture food products: identity of aquaculture farmer; harvest location (e.g., name of facility such as pond, pool, tank, etc.); date and duration of catch (harvest) time; quantity of catch/harvest. Life fish waterless transport: fish’s oxygen, carbon dioxide, glucose, and blood levels and temperature.	[11,47]
Processing	Responsible personnel; slaughterhouse location and slaughtering condition (e.g., in meat supply chain); processing technology and method; date of production; ingredient type and origin; batch and serial number of the product; packaging information; quality status; expiry date.	[11,14,23]
Storage (warehousing)	Warehouse number; entry and exit time; light; moisture; temperature; storage area quality; and other required information on the warehouse process.	[11,14,34]
Distribution (transporting)	Vehicle; personnel; transport route; cold chain information; delivery date; product ownership transfer.	[14,36,41]
Retailing	Responsible operator; receiving date and time; location, temperature, and humidity information from the retailer; product shelf time; nutritional value.	[14,23,36]
Consumption (e.g., restaurant; catering; home)	Receiving date; cooking time data; storage temperature and moisture before cooking; consumer complaint information.	[34,57]

**Table 3:** Some of the challenges in the application of BCT-based traceability systems in AFSCs.

Description of Challenges	References
Disruption of existing AFSCM methods. The AFSC and its traceability system are highly complex, and many stakeholders may resist such disruption.	[6,11,40,61]
Long AFSCs have many stakeholders with heterogeneous roles and demands and are more vulnerable to counterfeiting. This makes the application of BCT more difficult and less effective.	[9,13,22]
Establishing a robust consensus regarding data ownership, storage, and sharing among many actors (parties) in complex and global AFSCs is a challenge.	[9,14,16,21]
A BCT with a distributed ledger enables one to avoid data alteration once the data have entered the BCT. However, it cannot necessarily ensure the quality of data inserted into the blockchain.	[9,34]
Deciding on the type of BCT to be adapted (public, private, or permissioned) for a specific AFSC can be challenging and requires more pre-assessment work.	[9]
A BCT-based traceability system for the entire AFSC may demand considerable energy, personnel training, and digital infrastructure, leading to high investment costs.	[6,13,16,23,61]
In AFSCs that involve stakeholders from both low-income and high-income countries, there are challenges related to the cost and effectiveness of BC-based TSs due to the gap in digital infrastructures. This is one of the effects of the digital divide between industrialized and developing countries.	[26,62,63]
Developing a system with an adequate transaction processing capacity and the ability to store data directly in the blockchain is challenging.	[29]
Limitations on standards, data size, legal frameworks, and digital skills.	[3,11,13,57]
Difficulties in filtering out an individual company’s sensitive data to prevent this information from entering into the blockchain and being shared with others.	[11,13]
Slow uptake of BCT in AFSCs due to high investment costs and a lack of incentives for companies.	[61]
BCT has an anti-fraud and anti-“third-party-involvement” character. Therefore, resistance is expected from some users of BCT, and this reduces the rate of its implementation in many business sectors, including AFSCs.	[1,44]

**Table 4:** Some perceived benefits of BCT-based traceability systems in AFSCs.

Description of Benefits	Reference
No dependency on a central data server.	[9]
Enhanced data storage technology due to the use of a P2P distributed network with many participants (computers).	[11]
Reduces the risk of food safety incidents and related product recall costs and losses.	[11,13,29,65]
Less risk of data loss or hacking, as BCT enhances technological innovation that contributes to defense against cyber security attacks.	[9,11,16,29,66]
An automated system is used to timestamp food data and transactions and enhance the performance of the traceability system by increasing the efficiency, trust, and quality of the TS and the resilience of the AFSC.	[11,67]
Strengthens collaboration (cooperation) between AFSC actors connected as P2P network members.	[9,17,39,68]
BCT-based traceability enables one to establish a more secure and safe operational environment in the AFSC, with increased trust, efficiency, resilience, and food quality preservation and the prevention of fraud, counterfeiting, and the use of excessive preservatives.	[23,35,39,59,66,69]
The application of BC-based traceability can have positive effects on the sustainability of AFSCs.	[4,49]
BC technology is new, while BC-based traceability is immature and has potential for future research.	[9,70]
It promotes fair trade and enables one to make safe food purchase decisions.	[46,57,61]
BCT can be integrated into existing digital traceability systems.	[11,24,60]
Increases the competitiveness of FSC actors by enhancing the brand reputations of firms, which helps to attract more consumers and penetrate new domestic and international markets, e.g., meat product value chains.	[7,12,40,64,69]
BCT enables the development of closer relationships between producers and consumers and improves working conditions throughout the supply chain.	[61]
BCT can be used as a digital service platform that the supply chain actors can access and use to provide or obtain data regarding their products, and AFSC managers can audit the product flow easily.	[11,70]
There is potential for the design and implementation of BCT-based TSs at lower and affordable costs, e.g., management overhead costs can be shared among blockchain network parties.	[18]

## Data Availability

Data sharing not applicable.
